# Leakage of astrocyte-derived extracellular vesicles in stress-induced exhaustion disorder: a cross-sectional study

**DOI:** 10.1038/s41598-021-81453-8

**Published:** 2021-01-21

**Authors:** Johanna Wallensten, Anna Nager, Marie Åsberg, Kristian Borg, Aniella Beser, Alexander Wilczek, Fariborz Mobarrez

**Affiliations:** 1 Academic Primary Health Care Centre, Region Stockholm, Solnavägen 1E, Box 45436, 10431 Stockholm, Sweden; 2 Department of Clinical Sciences, Karolinska Institutet, Danderyd University Hospital, 18288 Stockholm, Sweden; 3 Division of Family Medicine and Primary Health Care, Department of Neurobiology, Care Sciences and Society, Karolinska Institutet, 14152 Stockholm, Sweden; 4 Department of Medical Sciences, Uppsala University, 75185 Uppsala, Sweden

**Keywords:** Neuroscience, Biomarkers, Diseases, Medical research, Neurology, Pathogenesis

## Abstract

Patients with stress-induced exhaustion disorder (SED) demonstrate cognitive dysfunction similar to patients with minor traumatic brain injury (TBI). We have previously detected elevated concentrations of astrocyte-derived extracellular vesicles (EVs) in patients with TBI. As such, we hypothesized that astrocyte-derived EVs could be higher in patients with SED than in patients with major depressive disorder (MDD) and healthy controls. Patients with SED (n = 31), MDD (n = 31), and healthy matched controls (n = 61) were included. Astrocyte-derived EVs (previously known as microparticles) were measured in plasma with flow cytometry and labeled against glial fibrillary acidic protein (GFAP) and aquaporin 4 (AQP4). In addition, platelet EVs and their CD40 ligand expression were measured. Patients with SED had significantly higher concentrations of AQP4 and GFAP-positive EVs and EVs co-expressing AQP4/GFAP than patients with MDD and healthy controls. Patients with MDD had significantly higher concentrations of GFAP-positive EVs and EVs co-expressing AQP4/GFAP than healthy controls. Platelet EVs did not differ between groups. CD40 ligand expression was significantly higher in patients with SED and MDD than in controls. In conclusion, the present study suggests that patients with SED, and to some extent, patients with MDD, have increased leakage of astrocyte-derived EVs through the blood–brain barrier.

## Introduction

Prolonged, unrelieved, intensive stress can lead to a state of chronic exhaustion that is characterized by profound fatigue, emotional instability, impaired sleep, and cognitive problems such as attentional and executive dysfunction. A century ago, the condition was called acquired neurasthenia and was typically thought to be caused by overwork. More recently, it has been called burnout, but since burnout usually refers to an emotional reaction to adverse work conditions^1^ rather than to an illness, this label appears to be a misnomer. In an attempt to differentiate more severe clinical illness, a growing number of researchers now use the term clinical burnout^2^. More recently, subject experts have introduced the term stress-induced exhaustion disorder (SED) (Table 1) and provided a description of the disorder’s main symptoms and course^3^. SED markedly impairs people’s capacity to work and function in daily life and is often associated with long periods of sick leave^4^.

**Table 1:** Criteria for stress-induced exhaustion disorder according to the Swedish National Board of Health and Welfare and Swedish version of the International Classification of Diseases, 10th edition (code F-43.8).

A. Physical and mental symptoms of exhaustion for at least two weeks. The symptoms have developed in response to one or more identifiable stressors present for at least 6 months
B. The clinical picture is dominated by markedly reduced mental energy, as manifested by reduced initiative, lack of endurance, or increased time needed for recovery after mental effort
C. At least four of the following symptoms have been present, nearly every day, during the same 2-week period: Concentration difficulties or impaired memory Markedly reduced capacity to tolerate demands or to work under time pressure Emotional instability or irritability Sleep disturbance Marked fatigability or physical weakness Physical symptoms such as aches and pains, palpitations, gastrointestinal problems, vertigo, or increased sensitivity to sound
D. The symptoms cause clinically significant distress or impairment in occupational, social, or other important respects
E. The symptoms are not due to the direct physiological effects of a substance (e.g. a drug of abuse, a medication) or a physical illness/injury (e.g. hypothyroidism, diabetes, infectious disease)

Criteria A through E must be fulfilled to diagnose stress-induced exhaustion disorder.

Many patients with SED are anxious and sad and display depressive symptoms^3^. Thus, SED is sometimes regarded as a variety of depression (e.g., exhaustion depression or job stress-induced depression). SED and major depressive disorder (MDD) share the main symptoms of cognitive dysfunction and fatigue^5^. However, patients with SED seldom experience general feelings of guilt, hopelessness, or suicidal ideation, and depressive symptoms are not their main complaint^6^. Moreover, the natural course and treatment outcome of SED and MDD appear to differ^7,8^.

The main symptoms of SED are similar to symptoms of minor traumatic brain injury (TBI), i.e., post-concussion syndrome. The cognitive impairments that patients with SED experience, such as impaired attention, executive function, and memory, may last for several months or even years^9^. Research has demonstrated structural changes in the medial prefrontal cortex, hippocampus, and amygdala of patients with SED^10^. However, an imaging study has found that after 1–2 years, the thinned prefrontal cortex of patients with SED returns to normal^11^. Structural brain changes in patients with SED are similar to those found in patients with early life traumas^12^ or post-traumatic stress disorder^13^ and to those seen in rats subjected to chronic stress^14^.

In several neurological disorders, such as TBI and neurodegenerative disorders, increased concentrations of extracellular vesicles (EVs) from astrocytes have been detected in peripheral blood or cerebrospinal fluid^15,16^. EVs, also known as microparticles, are released during cell activation and/or apoptosis and resemble their parent cells in that they expose similar cell-specific and activation markers^17^. Astrocytes, which are important regulators in the formation and maintenance of the blood–brain barrier (BBB)^18^, can also release EVs upon stimulation. In a previous study of patients with TBI, we found elevated concentrations of astrocyte-derived EVs expressing glial fibrillary acidic protein (GFAP) and water channel protein aquaporin 4 (AQP4)^16^. In light of the symptoms of brain dysfunction and structural changes in the brain in patients with SED, as well as our findings of astrocyte-derived EVs in patients with TBI, we hypothesized that concentrations of EVs of astrocytic origin are increased in the peripheral blood of patients with SED. We therefore examined whether astrocyte-derived EVs are found in the peripheral blood of patients with SED, and if so, whether concentrations of these EVs differ in patients with SED, patients with MDD, and healthy controls.

## Methods

### Study design and participants

The study included 31 patients with SED, 31 patients with MDD, and 61 healthy controls. Patients with SED and patients with MDD present overlapping symptoms, and patients with MDD were therefore used as clinical controls. Between 2014 and 2018, patients with SED or MDD treated at a psychiatric outpatient clinic in Stockholm were consecutively included in the study. The clinic specialized in treating health care professionals with symptoms of SED, depression, and anxiety. Since too few patients with MDD could be recruited from the clinic, additional patients with MDD were recruited from a public outpatient clinic in Stockholm in 2018.

Patients who were diagnosed with SED or MDD were asked to participate in the study by their physician, occupational therapist, or nurse. Inclusion criteria were ongoing SED (Table 1) or MDD, age 18 through 65 years, ability to understand Swedish, willingness to undergo 30 to 40 min of standardized testing, and diagnosis no more than three months prior to inclusion. Patients fulfilling diagnostic criteria for both SED and MDD were included in the study only if the physician considered the patient’s depressive symptoms secondary to the symptoms of SED, and in that case in the SED group. Patients with somatic or psychiatric diagnoses that could explain their symptoms better than SED or MDD were not included.

Physicians at the clinics diagnosed the patients with SED or MDD. The Mini-International Neuropsychiatric Interview (M.I.N.I) was used to exclude other psychiatric diagnoses. M.I.N.I was administered by a professional familiar with the instrument, either a clinic physician, clinic psychologist, or one of the researchers. Blood samples were obtained to exclude subclinical thyroid disease, anemia, vitamin B12 deficiency, or alcohol overconsumption.

Healthy controls were recruited in 2009 to provide blood samples to serve as reference material for future studies. At the request of the research group, Statistics Sweden, Sweden’s official government statistics agency, randomly selected 1146 permanent residents in the Stockholm area, aged 28 through 55, from the Swedish population register. The research group sent invitation letters to these people, and those who agreed to participate were contacted by phone for a first screening. People who reported that they were healthy were invited to an investigation that included a clinical and psychological examination. The investigation was performed by a physician. Those with current or previous SED, any other mental illness, endocrine or cardiovascular disorder, or cancer were excluded, resulting in 165 healthy controls who provided blood samples. Sixty-one of these 165 healthy controls, matched as closely as possible in age and sex to the SED and MDD groups, were selected for analysis of EVs.

The study was approved by the regional Ethical Review Board in Stockholm, Sweden (http://www.epn.se/en/start/) (Dnr. 2008/0061-31, 2014/585-31/1, 2016/1239-32, 2017/2088-32). All participants received verbal and written information about the study and provided written informed consent in accordance with the Declaration of Helsinki. All data were pseudonymized before linkage and analysis.

### Symptom rating scales

The self-assessment version of the Montgomery Asberg Depression Rating Scale (MADRS-S)^19^ was used to quantify the severity of depressive symptoms. It includes nine items rated on a scale from 0 to 6; higher scores indicate more severe symptoms. The 25-item Cognitive Failures Questionnaire (CFQ) was used to measure experience of cognitive failures in daily life, including failures of perception, memory, and motor function. Response alternatives range from 0 (never) to 4 (very often); higher scores indicate more frequent problems^20^.

### Sample collection

At inclusion, blood samples were obtained from patients with SED and MDD in accordance with a standardized protocol. All patients were asked to refrain from alcohol and physical exertion prior to blood sampling and to abstain from leaving a sample if they had signs of ongoing infection. Blood samples were drawn with a 21G sampling needle in the morning after overnight fasting, through direct venepuncture from an antecubital vein, after at least 15 min of rest. Samples were drawn into citrated tubes and centrifuged within 1 h at 2000*g* for 20 min at room temperature (RT), and subsequently stored at − 80 °C as platelet poor plasma. Blood samples for patients and controls were analyzed at Danderyd Hospital, Stockholm.

### Flow cytometric measurement of extracellular vesicles

Samples were thawed in a water bath at 37 °C for 5 min and subsequently centrifuged at 2000*g* for 20 min at RT. The upper supernatant was transferred to new tubes and centrifuged at 20 800*g* for 45 min at RT. The supernatant obtained by this second centrifugation step was discarded, and the EV-enriched pellet was used for the flow cytometric analysis. Subsequently, 20 µL of the pellet were incubated for 20 min in darkness, with 5 µL of anti-Aquaporin-4 Dylight 488 (corresponding to Human Aquaporin 4 aa 50–150, Abcam, Cambridge, UK), 5 µL anti-GFAP Dylight 755 (Abcam, Cambridge, UK), 5 µL CD41-FITC (Beckman coulter, Brea, CA, USA), and/or 5 µL CD154-PE (Beckman coulter, Brea, CA, USA). EVs were measured by flow cytometry on a Beckman Gallios instrument (Beckman coulter, Brea, CA, USA) with the threshold set to forward scatter. The EV gate was determined using Megamix-Plus FSC beads (0·3, 0·5 and 0·9 µm in size; BioCytex, Marseille, France). EVs were defined as vesicles less than 0·9 µm in diameter (forward scatter) and positive for the antibodies described above.

For the sake of brevity and convenience, in this study, microvesicles defined by their size and expression of GFAP and AQP4 (both single- and double-positive events were measured) are referred to as astrocyte-derived EVs. Both GFAP and AQP4 are markers of astrocytes^16^. AQP4 is a bidirectional water channel that is mainly found on astrocytes throughout the central nervous system^21^. However, GFAP has also been found in cells outside the central nervous system, such as lymphocytes^22^ and fibroblasts^23^.

**Figure 1:**
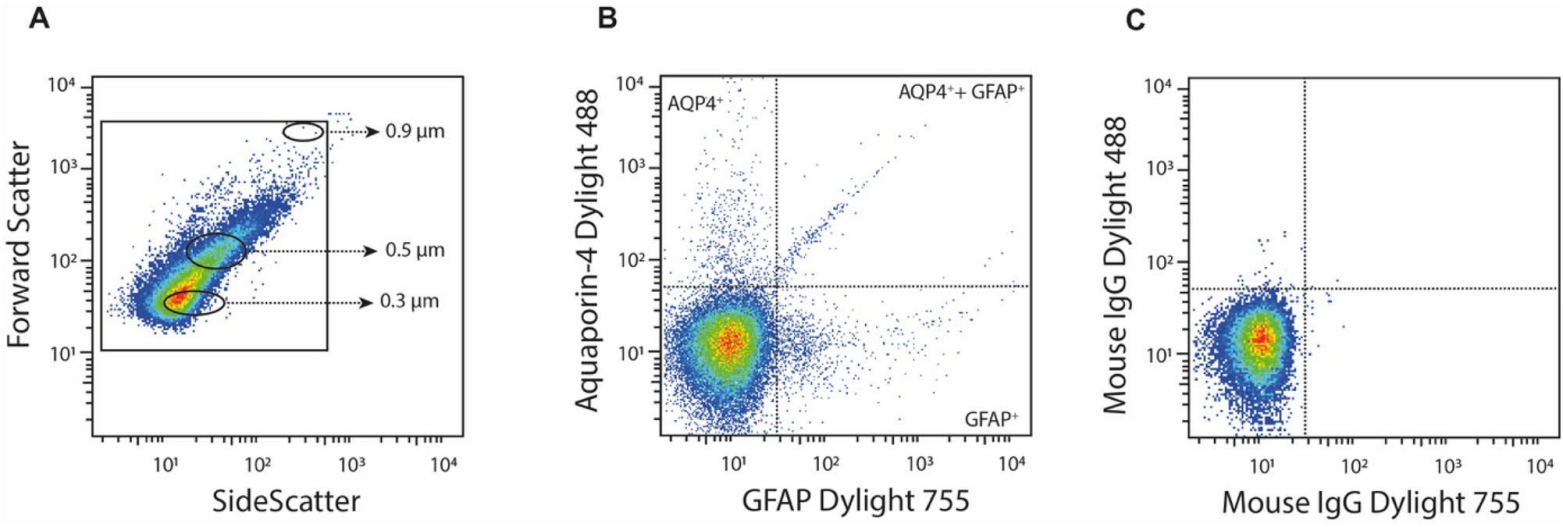
Flow cytometric analysis of astrocyte-specific extracellular vesicles in patients with stress-induced exhaustion disorder (SED). (**A**) Representative dot-plot of extracellular vesicle gating (based on size and complexity) together with gates demonstrating beads with a diameter of 0.3, 0.5, and 0.9 µm. (**B**) Representative dot-plot demonstrating aquaporin 4 (AQP4) and glial fibrillary acidic protein (GFAP) expression as well as vesicles exposing both AQP4 and GFAP. (**C**) Representative dot-plot demonstrating isotype mouse controls.

In addition to measuring astrocyte-derived EVs, we also measured the concentration of platelet-derived EVs (CD41) and their expression of CD154 (CD40 Ligand). Conjugate isotype-matched immunoglobulin with no reactivity against human antigens was used as a negative control to define the background noise in the cytometric analysis (Fig. 1). Results are presented as EVs/μL plasma, processed from the 20 μL pellet obtained after high-speed centrifugation. The intra- and interassay coefficients of the flow cytometric analysis, which was performed with 10 normal pool plasma samples (5 intra- and 5 interassays), were each less than 9·0%*.*

### Statistical methods

Non-parametric or parametric tests were used as appropriate to compare descriptive characteristics of groups. The Kruskal–Wallis test was used for non-parametric tests of three groups and the Mann–Whitney test for two groups. ANOVA was used for parametric tests of three groups and un-paired t-tests for parametric tests of two groups. Bonferroni was used as a post-hoc test of Kruskal–Wallis and ANOVA tests. Prior to statistical analysis, EV data were log transformed if necessary to obtain a normal distribution. Patients and controls were compared with the un-paired t-test after normalization. *P* values of ≤ 0·05 were considered significant. Statistical analysis was performed using SPSS Statistics (IBM SPSS Statistics for Windows, v 26.0. Armonk, NY: IBM Corp.) and JMP software (SAS Institute, v12.0, Cary, North Carolina, USA).

## Results

### Descriptive characteristics

A total of 31 patients with SED, 31 patients with MDD, and 61 healthy controls were recruited to the study. None were excluded because of subclinical thyroid disease, anemia, vitamin B12 deficiency, or alcohol overconsumption. There were no significant differences between the three groups in age, sex, BMI, or erythrocyte levels (Table 2). MADRS-S scores differed significantly between the groups in a manner consistent with the diagnoses (*P* = 0·001). That is, patients with MDD had the highest MADRS-S scores, and patients with SED had MADRS-S scores lower than those with MDD but higher than those of healthy controls. CFQ scores also differed significantly between patients with SED and patients with MDD (*P* = 0·025); the highest scores were observed in patients with SED. Blood sample concentrations of leukocytes, erythrocytes, and platelets were normal in all groups except for one outlier in the SED group who had an elevated platelet concentration (657 × 10^9^/L). Leukocyte concentration differed significantly between the three groups (*P* = 0·023), with higher levels in patients with MDD than in healthy controls. Platelet concentrations differed significantly between the three groups *(P* = 0·002), with higher concentrations in patients with either SED or MDD than in healthy controls.

**Table 2:** Descriptive characteristics of patients with stress-induced exhaustion disorder (n = 31), patients with major depressive disorder (n = 31), and healthy controls (n = 61).

Descriptive characteristics	Stress-induced exhaustion disorder (SED)	Major depressive disorder (MDD)	Healthy controls	*P* value
Mean age in years	n = 31 44·6 (9·7)	n = 31 40·3 (10·8)	n = 61 42·2 (9·5)	0·206
Women	n = 31 27 (87·1%)	n = 31 26 (83·9%)	n = 61 52 (85·2%)	0·999
Mean BMI	n = 31 24·8 (5·5)	n = 31 25·0 (5·0)	n = 61 24·7 (3·6)	0·898
Mean MADRS-S sum	n = 22 19·9 (5·6)	n = 25 27·1 (7·9)	n = 61 5·0 (3·6)	0·001*
Mean CFQ sum	n = 14 57·7 (11·0)	n = 26 50·0 (12·1)	n = 0	0·025**
Mean leukocyte concentration (10^9^/L)	n = 28 5·4 (1·3)	n = 28 5·9 (1·2)	n = 61 5·1 (1·2)	0·023***
Mean erythrocyte concentration (10^12^/L)	n = 28 4·5 (0·4)	n = 28 4·6 (0·4)	n = 0	0·610
Mean platelet concentration (10^9^/L)	n = 28 271·1 (92·0)	n = 28 251·8 (49·5)	n = 61 218·6 (26·4)	0·002*

Data are mean (SD) or n (%).

*Significant at a 0.05 level between SED and healthy controls and between MDD and healthy controls.

**Significant at a 0.05 level between SED and MDD.

***Significant at a 0.05 level between MDD and healthy controls.

24 patients with MDD, 25 patients with SED, and no healthy controls had antidepressant medication. There were no significant differences in concentrations of AQP4 and GFAP-positive EVs and EVs co-expressing AQP4/GFAP between patients with SED or MDD who received antidepressant medication (n=49) and patients with SED or MDD who did not receive such medication (n=10, missing data=3) (data not shown). In addition, there were no significant differences in the concentrations of leucocytes, erythrocytes, or platelets between patients with SED or MDD who received antidepressant medication (n=49) and patients with SED who did not receive such medication (n=10, missing data=3).

### Extracellular vesicles

**Figure 2:**
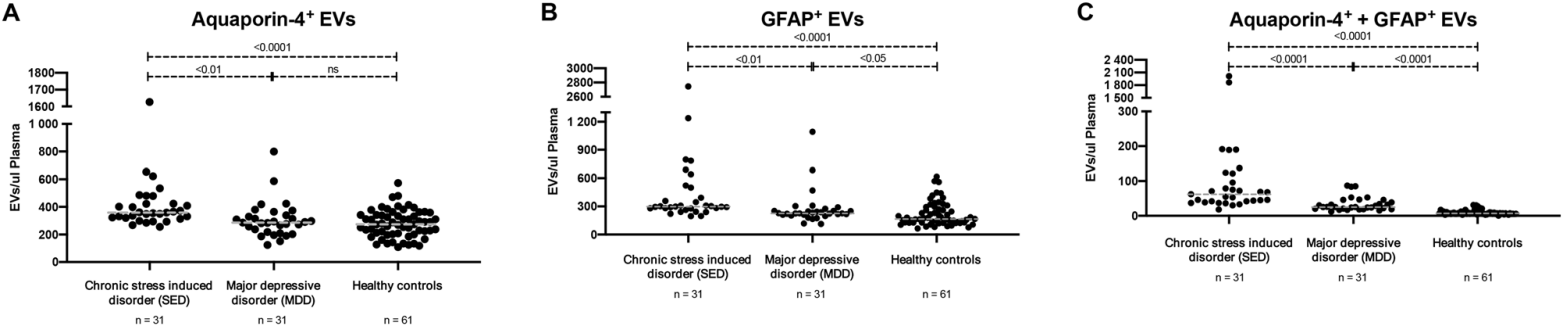
(**A**–**C**) Astrocyte-derived extracellular vesicles (EVs) expressing aquaporin 4 (AQP4) (**A**), glial fibrillary acidic protein (GFAP) (**B**), or both AQP4 and GFAP (**C**) in patients with stress-induced exhaustion disorder, patients with major depressive disorder, and healthy controls.

To detect astrocyte-derived EVs, we labeled the EVs with two astrocyte-specific markers, AQP4 and GFAP. Patients with SED had significantly higher concentrations of AQP4-positive EVs than patients with MDD (*P* < 0·001) and controls (*P* < 0·01) (Fig. 2A). GFAP-positive EVs followed the same pattern: higher concentration of GFAP-positive EVs were observed in patients with SED than either patients with MDD (*P* < 0·001) or healthy controls (*P* < 0·01) (Fig. 2B). We also investigated EVs that co-expressed AQP4 and GFAP, because such co-expression may provide a stronger indication that the EVs originate from astrocytes (Fig. 2C). Concentrations of EVs co-expressing AQP4 and GFAP were significantly higher in patients with SED than either patients with MDD (*P* < 0·001) or healthy controls (*P* < 0·01). Patients with MDD had significantly higher concentrations of both GFAP (*P* < 0·05) and EVs co-expressing AQP4 and GFAP (*P* < 0·001) than healthy controls.

**Figure 3:**
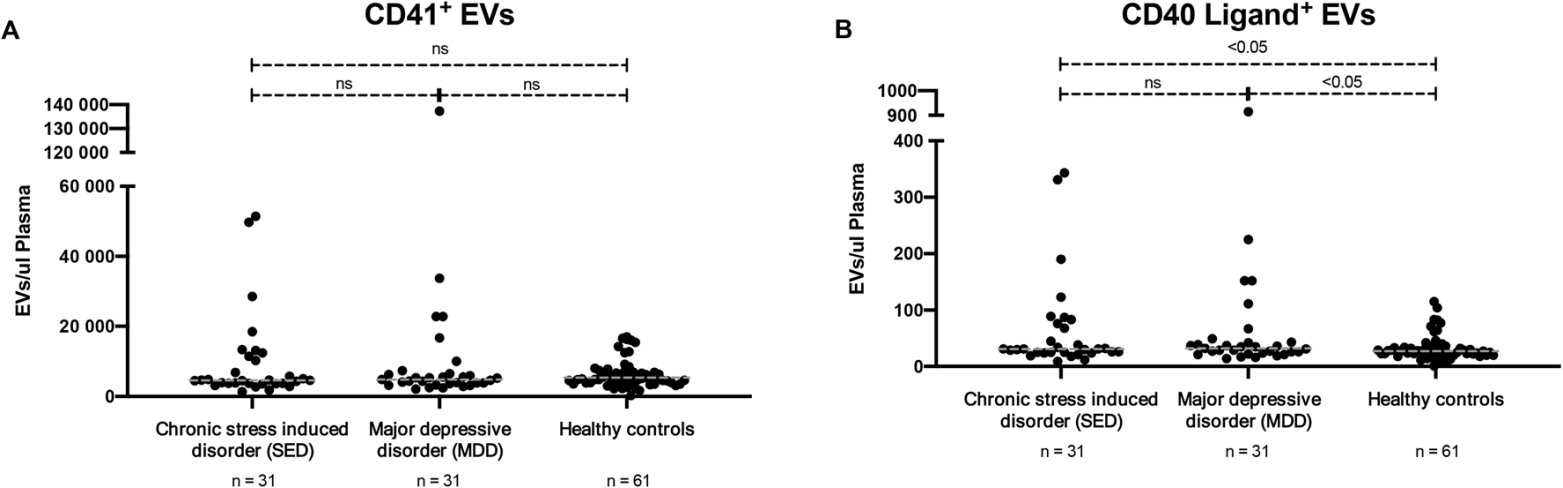
A and B. Extracellular vesicles (EVs) expressing platelet-specific marker CD41 (**A**) or CD40 Ligand (**B**) in patients with stress-induced exhaustion disorder, patients with major depressive disorder, and healthy controls.

In addition to astrocyte-derived EVs, we also investigated EVs originating from platelets (CD41^+^) and EVs expressing the inflammation marker CD154 (CD40 Ligand). There were no significant differences between groups in concentration of CD41-positive EVs (Fig. 3A). However, concentrations of EVs positive for CD40 Ligand were significantly higher in patients with SED and in patients with MDD than in healthy controls (*P* < 0.05) (Fig. 3B). No differences in CD40 Ligand expression were observed between patients with SED and patients with MDD (Fig. 3A,B). There was no correlation of clinical interest between concentrations of astrocyte-derived EVs and EVs positive for CD40 Ligand.

## Discussion

### Main findings

In this study, we observed EVs of astrocyte origin in the peripheral blood of patients with SED. Patients with SED had significantly higher concentration of astrocyte-derived EVs in their peripheral blood than patients with MDD and healthy controls. Thus, release and leakage of astrocyte-derived EVs through the blood–brain barrier resembles that observed after mechanical brain damage.

### Aquaporin-4 and glial fibrillary acidic protein

In the present study, we labeled astrocyte-derived EVs with two markers that can be elevated in patients with TBI^16^. One of these, AQP4, is a protein important for water and potassium homeostasis. It is predominantly found in the plasma membrane of astrocytes, concentrated in those domains facing vessels and pia mater, mediating the exchange of water between the brain and extracerebral liquids^21^. Changes in AQP4 expression and/or distribution have been found in neurological disorders such as amyotrophic lateral sclerosis, multiple sclerosis, and epilepsy, as well as in Alzheimer’s disease and in TBI^24^.

GFAP is a protein predominantly expressed by mature and reactive astrocytes. It appears to be an indicator of TBI; its plasma concentration has been correlated with intracranial trauma visible on CT scans and with CT-negative but MRI-positive findings^25^. Increased expression of GFAP has also been observed in the cerebrospinal fluid and in certain brain areas of people with neurodegenerative disorders, stroke, and malignant brain tumors^26^.

We also investigated platelet-derived EV concentrations and EVs expressing the inflammatory marker CD40 Ligand to observe potential changes in hemostatic and inflammatory parameters. Platelets are a major source of EVs in blood and are important in the development of inflammation^27^. We did not detect any increase in platelet-derived EVs, which suggests that release of astrocyte-derived EVs in patients with SED occurs independently of EVs that originate in blood. However, we observed slightly higher concentration of EVs that were positive for CD40 Ligand in patients with SED and MDD than in healthy controls. CD40 Ligand is released by both platelets and T-lymphocytes^28^. Further studies with larger sample sizes are needed to more fully investigate the role of T-lymphocytes in both SED and MDD.

### Astrocyte activation

Following mechanical brain damage, astrocytes activate to protect the structure and function of neurons and to demarcate brain tissue injuries. However, if not resolved in time, astrocyte activation and formation of glial scars may reduce neuroplasticity, and slow regeneration may negatively affect neuroplasticity and regeneration in the central nervous system (CNS)^29^.

Previous studies in rodents and postmortem studies in humans suggest brain region-specific alterations in astrocyte density and morphology in mood and stress disorders^30^. These changes could possibly lead to EV formation and release, as both cell activation and cell death lead to EV formation^17^.

Our finding of astrocyte-derived EVs in peripheral blood is in line with the findings of a 2019 study that detected GFAP and astrocytic protein aldolase C in the serum of rats exposed to stress^31^. Testing indicated that the aldolase C derived from the brain of the rats^31^.

### Blood–brain barrier permeability

The BBB protects the microenvironment of the CNS by restricting and controlling the passage of substances between the peripheral vascular system and the CNS. Astrocytes interact with endothelial cells of the BBB through astrocyte endfeet. In disorders characterized by mechanical CNS damage, such as brain injury and neurodegenerative disorders, it seems logical that leakage of astrocyte-derived EVs through the BBB could be measured in peripheral blood. However, the pathophysiological mechanism and thus the clinical relevance is yet to be discovered in patients with stress and mood disorders. Perhaps increased concentrations of astrocyte-derived EVs in peripheral blood, as seen in the present study and in the 2019 study of stress-exposed rats, result from increased permeability of the BBB. In stress and mood disorders, increased BBB permeability may enhance signaling in order to change behavior in the individual that supports recovery such as actions to decrease stress factors and increase time for rest.

**Figure 4:**
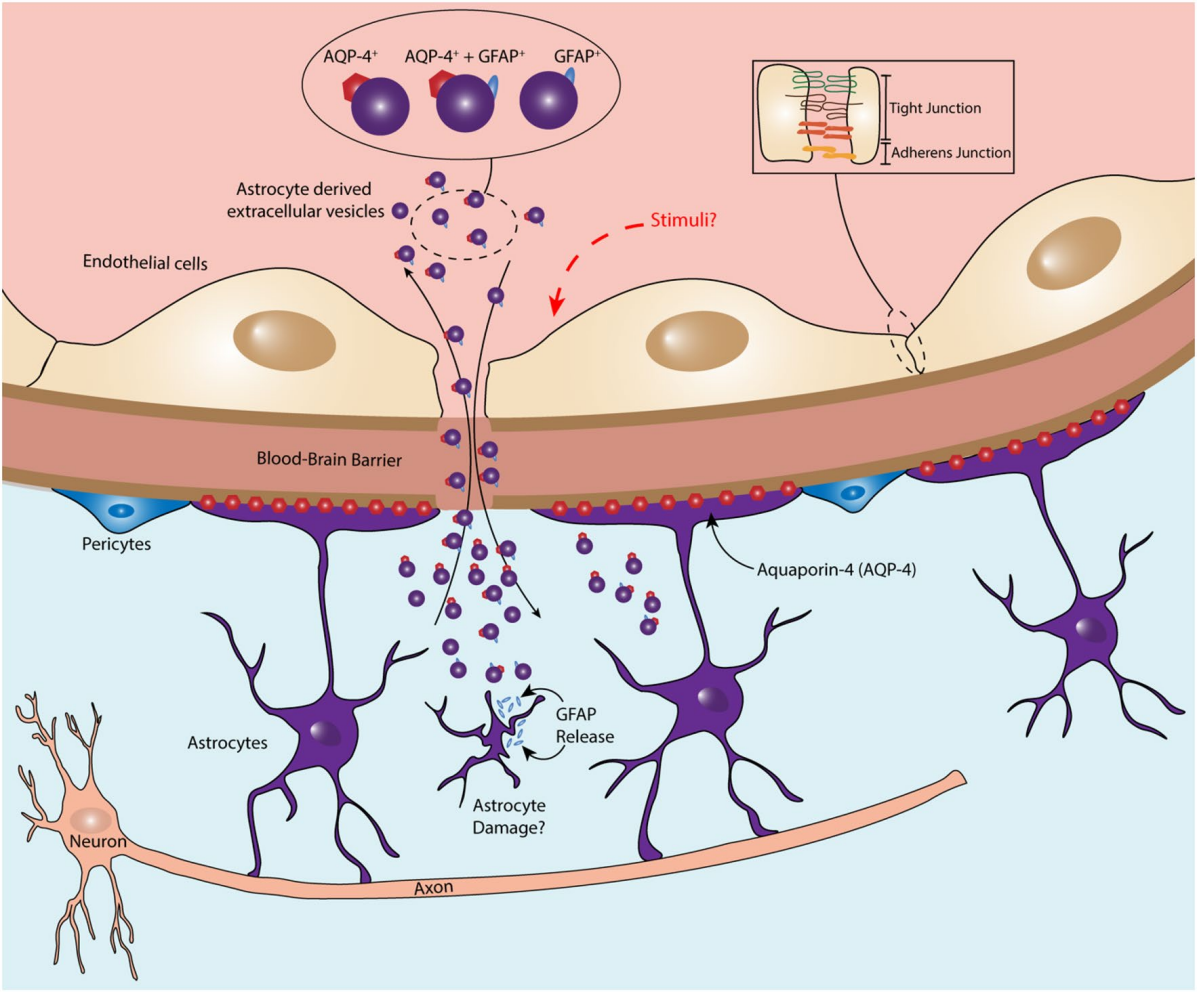
Our hypothesis is that stimuli can influence the blood–brain barrier to open the tight junction and adherent junction proteins, subsequently influencing astrocytes and leading to activation or apoptosis. The cellular changes cause the formation and release of extracellular vesicles, which could pass through the blood–brain barrier and be detectable in the blood.

Our hypothesis (Fig. 4) is that stimuli can influence the BBB to open the tight junction and adherent junction proteins. This could subsequently influence astrocytes, and depending on the type of stimuli, lead to activation or apoptosis. The cellular changes cause the formation and release of EVs, which could pass through the BBB and be detectable in the blood. The stimuli could be stress hormones, cytokines as part of inflammatory processes, and/or EVs from peripheral blood^32^. A study of children with obstructive sleep apnea found that EVs in peripheral blood disrupted the integrity of the BBB, especially in those with neurocognitive deficits^32^. Further studies are needed to better understand the processes that allow release of astrocyte-derived EVs from the CNS into peripheral blood after exposure to stress and the clinical relevance of this.

### Astrocyte-derived extracellular vesicles as biomarkers of stress-related disorders

We hypothesize that the increased plasma levels of astrocyte-derived EVs reflect stress-dependent structural changes in the brain occurring during, and possibly some time after, exposure to long-term stress without sufficient recovery and rest. These structural changes, and subsequently the release of astrocyte-derived EVs, are probably more pronounced in patients with SED than in patients with MDD since the symptoms in SED by definition are developed in response to stressors present for at least 6 months. MDD may also develop in response to stress, and being depressed is often stressful, however, MDD is not necessarily associated with long-term stress.

As such, the structural changes in the brain are probably associated with long-term cognitive impairment in patients with SED and may not be affected by antidepressant medication. Use of antidepressant medication did not predict the course of burnout symptoms in a study on patients with SED attending a stress clinic^6^.

The present study suggests that it is possible to detect these stress-dependent structural changes in the brain by measuring astrocyte-derived EVs in the peripheral blood. In order to use astrocyte-derived EVs as biomarkers, the finding needs to be replicated in larger studies, as well as patients in different stages of SED and with different mood and stress disorders. In the future, astrocyte-derived EVs might be useful as biomarkers for differential diagnosis and for measuring treatment effects in patients with stress-related disorders.

## Limitations

This study had several limitations. We collected blood samples from the patients during a different period than we collected them from the controls. However, blood sampling routines were the same in patients and controls, and the samples were analyzed in the same batches. AQP4 and GFAP are markers of astrocytes, but GFAP has also been found outside the central nervous system. It is therefore possible that EVs in the peripheral blood could have picked up GFAP from outside the central nervous system. We attempted to minimize this possibility by measuring for simultaneous presence of AQP4 and GFAP in the peripheral blood in all three groups. Further studies are needed to investigate the origin of the simultaneous presence of AQP4 and GFAP in peripheral blood in more detail. Furthermore, although the GFAP antigen we used is known to bind to GFAP protein, we cannot exclude the possibility that the antibody may have bound to something other than GFAP in our experiments. If unspecific binding were an issue, the concentration in healthy controls should have been higher as well, and it was not. From our study, it is clear that patients with SED and MDD have higher levels of AQP4 and GFAP exposed on EVs than healthy controls. Moreover, patients with SED and patients with MDD may have been in different stages of the diseases. To minimize this diversity, to be included in the study, patients with SED and patients with MDD had to be diagnosed less than 3 months before blood sampling, i.e., in a relatively early stage of the disorder. Lastly, the cross-sectional design makes it impossible to infer causality.

## Conclusions

In conclusion, patients with SED develop clinical features resembling brain dysfunction that are similar to clinical features in patients with minor traumatic brain injury. The present data demonstrate that patients with SED have higher concentrations of astrocyte-derived EVs in their peripheral blood than patients with MDD and healthy controls. Moreover, some subtypes of astrocyte-derived EVs are higher in both patients with SED and patients with MDD than in controls. The data therefore suggest that patients with SED, and to some extent, patients with MDD, have increased leakage of astrocyte-derived EVs through the BBB. Further studies are needed to investigate this mechanism in more detail as well as the potential to use astrocyte-derived EVs in diagnosing stress-related and mood disorders and in measuring treatment effects.

## Data Availability

The dataset generated and analyzed during the study is available from the corresponding author on reasonable request.
