# Author Correction: Leakage of astrocyte-derived extracellular vesicles in stress-induced exhaustion disorder: a cross-sectional study

**DOI:** 10.1038/s41598-023-37255-1

**Published:** 2023-06-23

**Authors:** Johanna Wallensten, Anna Nager, Marie Åsberg, Kristian Borg, Aniella Beser, Alexander Wilczek, Fariborz Mobarrez

**Affiliations:** 1 Academic Primary Health Care Centre, Region Stockholm, Solnavägen 1E, Box 45436, 10431 Stockholm, Sweden; 2 Department of Clinical Sciences, Karolinska Institutet, Danderyd University Hospital, 18288 Stockholm, Sweden; 3 Division of Family Medicine and Primary Health Care, Department of Neurobiology, Care Sciences and Society, Karolinska Institutet, 14152 Stockholm, Sweden; 4 Department of Medical Sciences, Uppsala University, 75185 Uppsala, Sweden

Correction to: *Scientific Reports* 10.1038/s41598-021-81453-8, published online 21 January 2021

The original version of this Article contained errors in the Methods and the Results sections.

In the Methods section, under the subheading ‘Study design and participants’,

“Between 2016 and 2018, patients with SED or MDD treated at a psychiatric outpatient clinic in Stockholm were consecutively included in the study.”

now reads:

“Between 2014 and 2018, patients with SED or MDD treated at a psychiatric outpatient clinic in Stockholm were consecutively included in the study.”

And,

“The study was approved by the regional Ethical Review Board in Stockholm, Sweden (http://www.epn.se/en/start/) (Dnr. 2014/585-31/1, 2016/1239-32 and 2017/970-21/1).”

now reads:

“The study was approved by the regional Ethical Review Board in Stockholm, Sweden (http://www.epn.se/en/start/) (Dnr. 2008/0061-31, 2014/585-31/1, 2016/1239-32, 2017/2088-32).”

In the Results section, under the subheading ‘Descriptive characteristics’,

“All patients with MDD and no healthy controls received antidepressant medication. There was no significant difference in EV concentration between patients with SED who received antidepressant medication (n = 5) and patients with SED who did not receive such medication (n = 26) (data not shown). In addition, there were no significant differences in the concentrations of leucocytes, erythrocytes, or platelets between patients with SED who received antidepressant medication (n = 5) and patients with SED who did not receive such medication (n = 26).”

now reads:

“24 patients with MDD, 25 patients with SED, and no healthy controls had antidepressant medication. There were no significant differences in concentrations of AQP4 and GFAP-positive EVs and EVs co-expressing AQP4/GFAP between patients with SED or MDD who received antidepressant medication (n=49) and patients with SED or MDD who did not receive such medication (n=10, missing data=3) (data not shown). In addition, there were no significant differences in the concentrations of leucocytes, erythrocytes, or platelets between patients with SED or MDD who received antidepressant medication (n=49) and patients with SED who did not receive such medication (n=10, missing data=3).”

The original Article has been corrected.

